# Mineralocorticoid Receptor Antagonists in Chronic Kidney Disease: Clinical Evidence, Pharmacology, and Drug–Drug Interactions for Personalized Management of Hyperkalemia

**DOI:** 10.3390/ijms27104272

**Published:** 2026-05-11

**Authors:** Toshinori Hirai, Kan Katayama

**Affiliations:** 1 Department of Pharmacy, Institute of Science Tokyo Hospital, 1-5-45 Yushima, Bunkyo-ku, Tokyo 113-8519, Japan; 2 Department of Cardiology and Nephrology, Mie University Graduate School of Medicine, 2-174 Edobashi, Tsu 514-8507, Japan

**Keywords:** aldosterone, mineralocorticoid receptor antagonist, hyperkalemia, potassium, chronic kidney disease, heart failure, pharmacokinetics, pharmacodynamics, drug–drug interaction

## Abstract

Mineralocorticoid receptor antagonists (MRAs) are the cornerstone of the management of heart failure and chronic kidney disease. A well-known adverse event, hyperkalemia, is associated with fatal arrhythmia and discontinuation of MRA. Our narrative review discusses the personalized treatment of MRAs, focusing on the pharmacological profile and drug–drug interactions to address safety concerns related to hyperkalemia. Clinicians should scrupulously monitor potassium levels, especially during dose titration, and review each patient’s medication list. Cytochrome P450 3A4 (CYP3A4) inhibitors are pharmacokinetic precipitators that interact with most MRAs, except spironolactone, and adversely affect the risk of hyperkalemia, although suggestive evidence is scarce. Potassium-elevating drugs synergistically increase serum potassium levels when co-administered with an MRA (e.g., renin-angiotensin aldosterone inhibitors, co-trimoxazole, non-steroidal anti-inflammatory drugs, calcineurin inhibitors, and β blockers). Additional approaches include correction of metabolic acidosis using sodium bicarbonate, potassium-lowering therapy using loop and thiazide diuretics, and sodium-glucose cotransporter 2 inhibitors. Novel potassium binders enable patients to receive the maximum-tolerated MRA with fewer gastrointestinal side effects. Individualized interventions for hyperkalemia risk are important in treatment using MRA.

## 1. Introduction

The prevalence of chronic kidney disease (CKD) driven by population aging is increasing worldwide [1]. CKD represents a major global burden on the healthcare system owing to its association with an increased risk of cardiovascular death and end-stage kidney disease, which increases the demand for renal replacement therapies, including dialysis and transplantation [2]. In addition to its impact on prognosis, CKD imposes a substantial financial burden, which increases along with disease severity [3,4]. Development of more effective therapeutic strategies for CKD is required to overcome these challenges.

CKD is defined by abnormality of kidney structure and/or function that persists for at least 3 months, typically characterized by an estimated glomerular filtration rate (eGFR) less than 60 mL/min/1.73 m^2^ [5]. In clinical practice, CKD is commonly classified by etiology and staged based on both eGFR and the urinary protein-creatinine ratio, especially the urinary albumin-creatinine ratio, in patients with diabetic kidney disease [6]. Decreased eGFR and increased urinary protein levels are well-known risk factors for adverse outcomes, including all-cause mortality, cardiovascular events, and CKD progression.

To reduce the risk of cardiorenal events in patients with CKD, pharmacological approaches have been established, including the use of renin–angiotensin–aldosterone (RAAS) inhibitors, sodium-glucose cotransporter 2 inhibitors (SGLT2i), and mineralocorticoid receptor antagonists (MRA). Among these therapeutic options, MRA has attracted considerable attention because of its cardiorenal protective effects, as demonstrated in recent clinical trials.

This narrative review aims to summarize the evidence and pharmacological mechanisms of MRA, emphasizing safety issues through pharmacological and pharmacokinetic considerations.

## 2. Clinical Evidence

Numerous double-blind randomized placebo-controlled trials have addressed the clinical benefits of MRA in patients with various cardiovascular and kidney conditions (Table 1). These studies will be pivotal in determining whether early introduction of MRA can fundamentally alter the natural history of cardiorenal events across various etiologies.

### 2.1. Spironolactone

The RALES trial demonstrated that the addition of 25 mg spironolactone to standard therapy, including angiotensin-converting enzyme inhibitors (ACEis), significantly reduced the risk of mortality and hospitalization in patients with a reduced left ventricular ejection fraction (LVEF) of <35% [7]. In the RALES trial, key exclusion criterion was a serum creatinine level exceeding 2.5 mg/dL. This trial demonstrated a significant increase in endocrinological outcomes such as gynecomastia, but not hyperkalemia, due to aldosterone blockade. A post hoc analysis of the RALES trial raised the alert over an increased risk of hyperkalemia, particularly, in patients with impaired kidney function and worsening renal function [8]. Additional analysis has suggested racial differences in the risk of hyperkalemia, with non-African American individuals (e.g., White and Asian) being more likely to develop hyperkalemia than African American individuals [9]. In contrast, the TOPCAT trial found no significant cardioprotective effect of spironolactone at doses of 15–45 mg in patients with heart failure and preserved ejection fraction (≥45%) (a median eGFR of 65.3 mL/min/1.73 m^2^) [10]. More recently, a randomized open-label trial reported no clinical superiority of spironolactone and a higher rate of treatment discontinuation due to reduced eGFR and an enhanced risk of hyperkalemia in older patients with CKD stage 3b (43.9 ± 6.9 mL/min/1.73 m^2^) and LVEF ≥ 40% [11].

### 2.2. Eplerenone

In the EMPHASIS-HF study, patients with LVEF of no more than 35% were randomly allocated to receive eplerenone up to 50 mg daily or a placebo in addition to standard therapy [12]. The findings suggested a significant reduction in cardiovascular mortality and morbidity. Similarly, in patients with heart failure following myocardial infarction, the EPHESUS trial showed the efficacy of eplerenone, titrated at 50 mg daily [13]. In contrast to the RALES trial, both trials identified hyperkalemia as an important safety concern, although an increased risk of gynecomastia was not observed. Notably, these study populations had relatively preserved renal function, with a mean eGFR of 71.2 ± 21.9 mL/min/1.73 m^2^ in the EMPHASIS-HF trial and a mean creatinine clearance of 79 ± 60 mL/min in the EPHESUS trial.

### 2.3. Finerenone

Several phase 3 trials have shown that finerenone reduces the risk of kidney and cardiovascular events in patients receiving maximally tolerated RAAS inhibitors, such as ACEi or angiotensin receptor blockers (ARBs), during the run-in period [14,15]. In the FIDELIO-DKD trial, the study participants had type 2 diabetes and CKD defined as either (1) a urinary albumin-creatinine ratio of 30 to 300 mg/gCr, eGFR of 25 to 60 mL/min/1.73 m^2^, and diabetic retinopathy, or (2) a urinary albumin-creatinine ratio of 300 to 5000 mg/gCr and eGFR of 25 to 75 mL/min/1.73 m^2^ [14]. The FIGARO-DKD trial confirmed cardioprotective effects of finerenone in patients with type 2 diabetes and CKD, using the following inclusion criteria: (1) a urinary albumin-creatinine ratio of 30 to 300 mg/gCr and eGFR of 25 to 90 mL/min/1.73 m^2^ or (2) a urinary albumin-creatinine ratio of 300 to 5000 mg/gCr and an eGFR of more than 60 mL/min/1.73 m^2^ [15]. Across these trials, hyperkalemia was consistently observed as the most frequent adverse event associated with finerenone use. The FINEARTS-HF study showed that finerenone significantly reduced the worsening heart failure in patients with preserved LVEF ≥ 40% and a mean eGFR of 61.9 ± 19.4 mL/min/1.73 m^2^, although hyperkalemic events were more frequent compared with placebo [16].

### 2.4. Esaxerenone

Esaxerenone has been approved for refractory hypertension in Japan and was under regulatory review in the United States and Australia at the time of manuscript preparation. The ESAX-HTN study was designed to randomly compare esaxerenone at 2.5 or 5.0 mg with 50 mg of eplerenone in hypertensive patients without CKD (<60 mL/min/1.73 m^2^). These findings revealed significantly lower blood pressure and comparable hyperkalemic risk between groups [17]. A prospective interventional study (EAGLE-DH), rather than a randomized design, evaluated the effect of the addition of esaxerenone to SGLT2 inhibitors for the management of patients with diabetes mellitus and hypertension (eGFR ≥30 mL/min/1.73 m^2^), suggesting significant decreases in blood pressure and urinary protein levels without an increased risk of hyperkalemia, regardless of the CKD stage [18].

## 3. Pharmacological Insights

### 3.1. Chemical Structures and Receptor Selectivity

MRAs are broadly classified into steroidal and non-steroidal skeletons based on their chemical structure. Steroidal MRAs include spironolactone and eplerenone; nonsteroidal MRAs include esaxerenone and finerenone [19]. The mineralocorticoid receptor is a member of the nuclear steroid receptor family, functioning as a ligand-activated transcription factor. Upon binding to its primary ligand, aldosterone, mineralocorticoid regulates the expression of genes involved in electrolyte and fluid homeostasis.

MRAs show different affinities for the androgen and progesterone receptors because of their structural similarity to endogenous steroid hormones. The traditional MRA spironolactone blocks the mineralocorticoid receptor and exhibits off-target binding to the androgen receptor as an antagonist, and the progesterone receptor as a partial agonist [20]. In contrast, nonsteroidal MRA (finerenone and esaxerenone) possess bulky structures, resulting in higher selectivity for mineralocorticoid receptors and minimal affinity for sex hormone receptors [21,22].

In vitro studies have demonstrated higher binding affinities of esaxerenone and finerenone for mineralocorticoid receptors than those of conventional steroidal MRAs. Finerenone shows high receptor selectivity with a half maximal inhibitory concentration (IC_50_) of 18 nM (spironolactone: 24 nM and eplerenone: 990 nM) and less affinity for other receptors at concentrations up to 10 μM [22,23]. Similarly, esaxerenone inhibits aldosterone binding with an IC_50_ of 9.4 nM, compared with 36 nM for spironolactone and 713 nM for eplerenone [24].

### 3.2. Mechanism of Action

**Figure 1:**
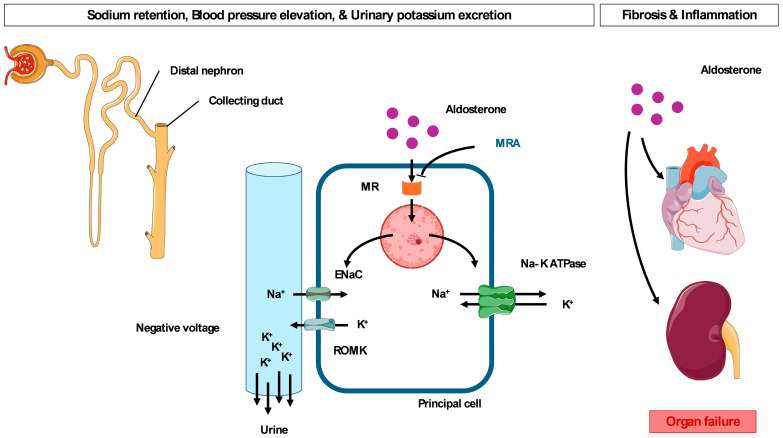
Pharmacological mechanism of mineralocorticoid receptor antagonists. Abbreviations: MR, mineralocorticoid receptor; MRA, mineralocorticoid receptor antagonists; ENaC, epithelial sodium channel; Na-K ATPase, sodium-potassium ATPase; ROMK, renal outer medullary potassium channel.

Mineralocorticoid receptors are primarily expressed in the principal cells of the distal nephron and collecting duct [25,26,27,28]. Upon aldosterone binding, the activation of mineralocorticoid receptors stimulates the transcription of the epithelial sodium channel (ENaC) and sodium-potassium ATPase (Na-K ATPase) (Figure 1).

Sodium enters principal cells via ENaC and is transported into blood vessels through the exchange of sodium and potassium via Na-K ATPase, which generates a negative lumen voltage [29]. This potential difference is offset by driving potassium secretion through the renal outer medullary potassium channel (ROMK) as a recycling route [30]. Overall, these physiological responses contribute to extracellular volume expansion, elevated blood pressure, and urinary potassium excretion. Therefore, aldosterone plays an important role in handling the potassium homeostasis aspect of urinary excretion by regulating ion channels.

Beyond electrolyte regulation, excess aldosterone promotes inflammation and fibrosis in both the heart and kidney by activating mineralocorticoid receptor signaling in cardiomyocytes and vascular smooth muscle [31,32,33]. Mineralocorticoid receptor signaling results in multiple pathways, including fibroblast proliferation and differentiation, oxidative stress, and endothelial dysfunction independent of blood pressure [34,35]. These effects are crucial in cardiac and renal remodeling, and ultimately worsen clinical outcomes.

Accordingly, MRAs exert cardioprotective effects through hypotensive effects and anti-inflammatory and antifibrotic effects, which support their clinical benefits in patients with heart failure and CKD [7,12,15,16].

### 3.3. Mechanism of Adverse Events

#### 3.3.1. Hyperkalemia

Hyperkalemia is the most common adverse event associated with the pharmacological mechanism of MRA [36]. A serum potassium level >6.0 meq/L is known to cause serious electrocardiographic changes, which leads to life-threatening arrythmia [37]. Because MRAs potentially pose a risk of fatal hyperkalemia, defined as serum potassium level more than 6.0 meq/L, especially in patients with a reduced kidney function [38], the risk of death associated with hyperkalemia should not be overlooked. This adverse event is a clinical barrier to the optimization of MRA therapy. MRAs increase serum potassium levels by inhibiting aldosterone-mediated potassium excretion.

A systematic review of randomized trials reported an approximately doubled incidence rate of hyperkalemia, defined as >5.5 meq/L, in patients with heart failure receiving MRAs compared to those receiving placebo (14% vs. 6.0%) [39]. Similar findings with an approximately two-fold increase in the pooled risk ratio of hyperkalemia following MRA treatment in patients with diabetic kidney disease and in those not requiring dialysis have been reported [40].

The occurrence of hyperkalemia often leads to withdrawal of MRA, ACEi, and ARB, potentially resulting in suboptimal treatment and worsening clinical outcomes, while alternatively avoiding recurrent episodes of hyperkalemia [41,42,43,44].

#### 3.3.2. Endocrine Adverse Effects

From a pharmacological perspective, spironolactone, a traditional nonselective MRA, elicits endocrine-related adverse effects, such as gynecomastia and irregular menstruation, through its action on androgen and progesterone receptors [45,46]. A meta-analysis implied that spironolactone, but not eplerenone, dramatically increased the risk of gynecomastia, with a relative risk of 7.37 compared with placebo [47]. In contrast, nonsteroidal MRAs were not associated with clinically significant endocrine adverse events, such as gynecomastia, because of their high receptor selectivity [48].

## 4. Pharmacokinetics and Drug–Drug Interactions

### 4.1. Pharmacokinetic Profile and Principle

Each MRA exhibits a different pharmacokinetic profile (Table 2) [49,50,51,52,53,54,55,56,57,58].

All MRAs are predominantly cleared via hepatic metabolism with minimal renal excretion of unchanged drugs and active metabolites. Except for spironolactone, MRA undergoes cytochrome (CYP) 3A4 metabolism and, to a lesser extent, CYP2C8 metabolism, particularly finerenone. In contrast, spironolactone is extensively converted to the active metabolite canrenone, primarily through non-CYP-mediated pathways. The systemic exposure can be described using the following equation:

$$AUC=\frac{F\times Dose}{{CL}_{h}}=\frac{F\times Dose}{{f_{u}\times CL}_{inth}}$$

where AUC is the area under the plasma drug concentration-time curve, F is the absolute bioavailability, and CL_h_ is the hepatic clearance. Under oral administration, CL_h_ can be approximated as a function of the unbound fraction (f_u_) and the hepatic intrinsic clearance (CL_inth_).

$${CL}_{h}={f_{u}\times CL}_{inth}$$

The area under the unbound plasma drug concentration-time curve (AUC_f_) can be expressed as

$${AUC}_{f}=\frac{F\times Dose}{{CL}_{h}/f_{u}}=\frac{F\times Dose}{{CL}_{inth}}$$

Alterations in intrinsic clearance, particularly those mediated by CYP enzymes, directly reflect exposure to pharmacologically active free drugs and the potential for drug–drug interactions, with particular clinical relevance in patients receiving multiple concomitant medications. Theoretically, changes in protein binding, due to hypoalbuminemia or the accumulation of uremic toxins, can be ignored regarding their impact on the pharmacological effect. The reason for this is that the steady-state unbound concentration is characterized primarily by CL_inth_ rather than f_u_. Consequently, even if f_u_ increases, the subsequent increase in the total clearance compensates for the change, leaving the unbound systemic exposure unchanged. Therefore, the impact of altered protein binding on systemic exposure is theoretically negligible. This review focused on systemic exposure and clearance-related characteristics, which are more directly relevant to drug–drug interactions in terms of CYP3A4 activity.

### 4.2. Pharmacokinetic Drug-Drug Interaction

Evidence for pharmacokinetic drug–drug interactions of spironolactone is limited, as it undergoes complex metabolism rather than metabolism through a CYP-dependent pathway. However, a meticulous understanding of the molecular mechanisms underlying drug–drug interactions is imperative for the safe administration of MRAs.

Strong CYP3A4 inhibitors raise concerns about the adverse events associated with eplerenone among azole antifungals [59]. A case report indicated lethal hyperkalemia resulting from the interaction of eplerenone with ritonavir in a patient infected with human immunodeficiency virus [60]. Hypotension has been reported following the coadministration of eplerenone and voriconazole in patients undergoing antihypertensive therapy, including nifedipine (a CYP3A4 substrate) [61].

A clinical study showed that itraconazole, administered at a loading dose of 200 mg twice daily followed by a dose of 200 mg once daily, increased systemic exposure to esaxerenone by approximately 1.5-fold, whereas the CYP3A4 inducer rifampicin at a dose of 600 mg once daily decreased esaxerenone exposure by approximately 70% [62]. In real-world data, co-prescription of clarithromycin, a macrolide antibiotic, has no additional risk of hyperkalemia associated with eplerenone or esaxerenone. However, the magnitude of serum potassium elevation correlates positively with age among patients receiving eplerenone or esaxerenone and clarithromycin [63].

As finerenone is predominantly metabolized by the CYP3A4 isoenzyme, co-administration of moderate-to-strong inhibitors can drastically alter its pharmacokinetic profile. Clinical pharmacokinetic studies have reported that co-administration of finerenone with moderate CYP3A inhibitors, such as erythromycin and verapamil, increased the AUC by 3.48- and 2.70-fold, respectively [56,64,65]. Pharmacokinetic simulations support these interactions between finerenone and CYP3A4 modulators [66]. However, clinical evidence regarding drug interactions for finerenone using real-world data remains limited compared to steroidal MRAs. Further analysis is required to ensure safety in diverse global populations.

The pharmacokinetic diversity among MRAs has direct implications for clinical safety. The extensive reliance on the CYP3A4 pathway makes systemic exposure vulnerable to alterations by concomitant CYP3A4 inhibitors. Accordingly, this phenomenon necessitates a meticulous review of the medication lists to prevent drug overexposure and subsequent adverse events.

### 4.3. Pharmacodynamic Drug-Drug Interaction

Combination therapy with antihypertensive drugs is widely prescribed in clinical practice and often results in enhanced hypotensive effects. In this review, we discuss the pharmacodynamic interactions, focusing on hyperkalemia, a common side effect associated with MRAs.

#### 4.3.1. RAAS Inhibitors

The RAAS pathway is key to potassium excretion in urine. Therefore, the addition of MRAs to RAAS inhibitors (ACEi and ARB) dramatically increases the risk of hyperkalemia compared with RAAS inhibitor monotherapy [67]. In particular, the combination of ACEi and ARB failed to show a prognostic benefit in populations at high risk of cardiovascular diseases, posing a negative impact on the risk of both acute kidney disease and hyperkalemia [68,69]. Dual RAAS inhibition (ACEi plus ARB) combined with an MRA should be avoided because of the risk of hyperkalemia.

#### 4.3.2. Trimethoprim

Co-trimoxazole consists of trimethoprim and sulfamethoxazole at a fixed ratio of 1:5 [70]. Trimethoprim competitively inhibits ENaC, causing hyponatremia due to renal salt wasting and hyperkalemia due to reduced urinary potassium excretion [71,72,73]. Co-trimoxazole is a risk factor for hyperkalemia-related hospitalization and cardiac arrest in older patients receiving spironolactone [74,75]. A dose-dependent effect on hyperkalemia risk has been observed in the cohort of patients treated with co-trimoxazole [76,77]. An elevation in serum potassium levels may be explained by trimethoprim dose-corrected by eGFR excreted via the kidney, after co-administration of esaxerenone and co-trimoxazole [78].

#### 4.3.3. Non-Steroidal Anti-Inflammatory Drugs (NSAIDs)

NSAIDs inhibit renin secretion and constrict afferent arterioles by blocking prostaglandin synthesis, leading to reduced eGFR [79,80,81]. The triple combination of RAAS inhibitors, NSAIDs, and diuretics is referred to as a “triple whammy” [82], a well-recognized harmful combination that can lead to acute kidney injury and subsequent hyperkalemia [83]. An epidemiological study suggested the association of early onset acute kidney injury with NSAID prescription [84]. Moreover, an increased risk of acute kidney injury has been observed with dual or triple therapies, including diuretics [85]. To avoid unexpected hyperkalemia associated with MRAs, it is imperative for clinical specialists to pay attention to co-administration of NSAIDs.

#### 4.3.4. Calcineurin Inhibitors

Calcineurin inhibitors are immunosuppressive drugs that prevent graft loss after transplantation [86]. The combination of spironolactone and calcineurin inhibitors can accelerate the decrease in eGFR and increase potassium levels in kidney recipients [87]. There was a notable difference in the risk of hyperkalemia between calcineurin inhibitors. Tacrolimus is associated with a greater increase in potassium levels than cyclosporine [88]. In the available data on tacrolimus, a higher elevation in potassium was observed at a high trough concentration than at a low trough concentration (median [interquartile range]: 11.3 [9.2 to 13.8] ng/mL versus 8.9 [7.3 to 10.5] ng/mL) [89]. Tacrolimus has large pharmacokinetic variability originating from congenital and acquired factors, such as CYP genotype, drug interactions, and chronic inflammatory disorders, including diabetes [90,91,92,93]; therefore, therapeutic drug monitoring is useful to mitigate the concentration-dependent hyperkalemic effect of tacrolimus.

#### 4.3.5. β-Blockers

β-blockers inhibit renin release and may reduce cellular potassium uptake, thereby increasing the risk of hyperkalemia [94]. Hyperkalemia is more frequently observed in patients receiving non-selective β-blockers than cardioselective β-blockers [95]. When prescribing β-blockers for cardioprotection, bisoprolol (a cardioselective β-blocker) may be preferred to mitigate hyperkalemia. A sub-analysis of FIDELIO-DKD identified co-administration of a β-blocker as a risk factor for hyperkalemia [96]. Data from a registry database suggested that the greatest risk of hyperkalemia occurs with the combination of RAAS inhibitor, β-blocker, and MRA within 90 days [97].

## 5. Safety Issues

From the perspective of safety, hyperkalemia is a life-threatening adverse event associated with MRA use because severe hyperkalemia causes ventricular tachycardia and sudden cardiac death [37,98,99]. The fear of life-threatening hyperkalemia might be associated with clinical inertia. Addressing this safety concern through a rigorous understanding of pharmacological mechanisms and evidence-based mitigation strategies is essential for providing MRA therapy in real-world patients. We discuss the management of hyperkalemia in patients undergoing MRA.

Several randomized trials have rigorously prespecified the exclusion of patients treated with CYP3A4 inhibitors, such as azole antifungals [12,14,15,16]. Despite the large dataset, the understanding of hyperkalemic risk considering the impact of drug interaction is limited, including CYP3A4 inhibitors, except for frequent use of medications such as RAAS inhibitors and β-blockers [96,100,101]. In a classical randomized study, the RALES trial found no significant increase in the risk of hyperkalemia. However, subsequent real-world data demonstrated a marked increase in the rate of hyperkalemia-related hospitalization and mortality following the prescription of spironolactone after the publication of the RALES trial [102]. In the real-world population, drug–drug interactions can substantially modify the efficacy and toxicity of drugs through pharmacokinetic and pharmacodynamic changes. Thus, hyperkalemia should be managed considering these factors as listed in Table 3.

### 5.1. Monitoring

Many studies have reported suboptimal monitoring of serum potassium levels [103,104,105]. For instance, serial potassium and kidney function tests should be performed at one week, four weeks, and every six months after the introduction or titration of an MRA [106]. The kidney is the primary organ responsible for potassium excretion, accounting for approximately 90% of daily potassium elimination. Importantly, renal function is the principal determinant of potassium homeostasis, and impaired renal excretion is the most significant risk factor for hyperkalemia, especially in patients with CKD. Mitigating the risk of hyperkalemia requires not only clinical monitoring but also proactive patient education. In addition to monitoring, medical practitioners should provide clear guidance on dietary potassium intake, particularly when MRAs are combined with other RAAS inhibitors. Furthermore, patients must be cautioned against the use of over-the-counter drugs such as potassium preparation and NSAIDs, which can precipitate subsequent risk of hyperkalemia. Empowering patients with this knowledge is essential for the long-term management of MRAs.

### 5.2. Medication Review

**Figure 2:**
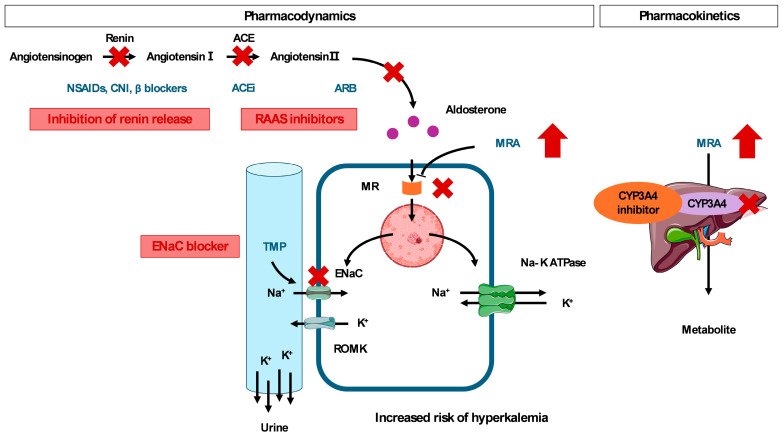
Drug-drug interaction regarding mineralocorticoid receptor antagonists. Abbreviations: NSAIDs, non-steroidal anti-inflammatory drugs; CNI, calcineurin inhibitors; ACE, angiotensin-converting enzyme; ACEi, angiotensin-converting enzyme inhibitor; ARB, angiotensin-receptor blockers; RAAS, renin–angiotensin–aldosterone; TMP, trimethoprim; MR, mineralocorticoid receptor; MRA, mineralocorticoid receptor antagonist; ENaC, epithelial sodium channel; Na-K ATPase, sodium-potassium ATPase; ROMK, renal outer medullary potassium channel; CYP, cytochrome P450.

To avoid hyperkalemia, a step-by-step approach is advantageous for optimizing MRA treatment, particularly in patients with CKD [107]. Through a comprehensive review of medication, clinicians should screen for drugs that lead to hyperkalemia or require MRA dose adjustments (Figure 2).

As described under “Pharmacokinetics and Drug–Drug Interactions”, concomitant precipitating drugs should be avoided whenever possible or used with caution. Overall, appropriate monitoring and medication review strategies are essential to minimize the risk of adverse events and continue the use of MRAs in clinical practice. This comprehensive medication review should not be a one-time event but rather a continuous process integrated into every clinical encounter. Proactive communication between healthcare providers and patients ensures that any new prescriptions or over-the-counter medications are evaluated for potential interactions that could jeopardize the safety of MRA therapy.

### 5.3. Correction of Metabolic Acidosis

Metabolic acidosis worsens the exchange of protons and potassium, thereby suppressing the extracellular shift of potassium. A randomized study demonstrated that sodium bicarbonate at a dose of 0.4 meq/kg of ideal body weight results in improved bicarbonate levels and reduced potassium levels in CKD stages 3 and 4 [108]. Another trial has shown a numerical difference in serum potassium level between doses of sodium bicarbonate (0.8 meq/kg of lean body weight versus 0.5 meq/kg of lean body weight) in patients with baseline eGFR of 36 ± 9 mL/min/1.73 m^2^ [109]. The collected evidence suggests that sodium bicarbonate may alleviate the risk of MRA-induced hyperkalemia.

### 5.4. Potassium-Lowering Strategy

Loop and thiazide diuretics pharmacologically promote the urinary excretion of potassium [110]. These agents are generally co-administered to control blood pressure and fluid volume. As a consequence of diuretic therapy, volume depletion adversely affects acute kidney injury and worsens hyperkalemia. Follow-up of kidney function and diuretic titration are important for managing hyperkalemic events.

SGLT2i lowers potassium as a pleiotropic effect underlying the varying mechanisms of increased sodium delivery and osmotic diuretics due to glycosuria [111]. This effect acts to create a more favorable electrochemical gradient for potassium secretion via the ROMK channels. This compensatory mechanism effectively offsets the potassium-elevating effect of MRAs, potentially expanding the therapeutic window for patients who were previously considered intolerant to MRAs due to hyperkalemia risk. Indeed, a meta-analysis suggests that SGLT2i mitigates the risk of hyperkalemia in patients with heart failure undergoing MRA treatment [112]. In contrast, SGLT2i exerted a neutral effect on serious hyperkalemia in a subpopulation of patients with type 2 diabetes receiving MRAs [113]. For patients deemed suitable for SGLT2i, its introduction may provide a benefit by minimizing the risk of hyperkalemia and exerting a cardiorenal protective effect.

Potassium binders directly capture potassium through cation exchange, impairing its absorption in the gut [114]. Conventional potassium binders (polystyrene resins) can cause adverse gastrointestinal events such as constipation, ischemic colitis, and bowel necrosis [115]. Novel potassium binders such as sodium zirconium cyclosilicate and patiromer are better tolerated, with a rapid and sustained hypokalemic effect [116]. Recently, new potassium binders have allowed the continuation of treatment with RAAS inhibitors by avoiding episodes of hyperkalemia in patients with hypertension, heart failure, and CKD [117,118,119]. Future investigations should focus on whether the co-administration of novel potassium binders can safely extend the use of MRAs to vulnerable population, thereby bridging the current gap in evidence-based management.

## 6. Future Perspectives

Regrettably, a medication review is a posterior approach. Although preemptive medicine has attracted attention, this concept has not yet been implemented in the field of drug-induced electrolyte abnormalities. Using a pharmacometrics approach, the risk of hyperkalemia in patients receiving co-trimoxazole can be predicted [120,121]. This analysis demonstrated that the risk of hyperkalemia is significantly associated with high-dose trimethoprim and baseline potassium level. A pharmacometric model has been used to evaluate the magnitude of blood concentration changes owing to drug interactions with tacrolimus and a couple of CYP3A4 inhibitors of voriconazole and clarithromycin [122]. This knowledge can be expanded to risk prediction for hyperkalemia associated with MRAs. Specifically, by incorporating specific parameters such as the dose–response relationship of potassium elevation, renal function, and the inhibitory effect on metabolic enzymes (e.g., CYP3A4) into a pharmacometric framework, clinicians can simulate the risk of hyperkalemia for individual patients. This approach justifies treatment feasibility and informs precise dose adjustments based on clinical backgrounds and potential drug interactions, shifting the management of MRA therapy for a preemptive strategy.

Furthermore, the integration of digital health tools and remote monitoring systems could revolutionize the safety of MRA therapy. Real-time potassium monitoring, coupled with risk prediction models that incorporate pharmacokinetic and pharmacodynamic data, holds the potential to proactively identify patients at risk of adverse events before clinical complications occur. Such technological advancements will be instrumental in the preemptive management of hyperkalemia associated with MRAs.

Finally, a multidisciplinary approach involving nephrologists, cardiologists, and pharmacists is essential to optimize MRA treatment. Collaborative medication reviews and patient education are key to ensuring long-term adherence and minimizing the risk of preventable adverse drug interactions.

## 7. Conclusions

We have comprehensively reviewed safety aspects of MRA associated with hyperkalemia in CKD. Several studies suggest that MRAs can improve unfavorable cardiorenal outcomes. Given the underlying mechanism of hyperkalemia and the inter-subject variability in pharmacotherapy, MRA treatment should be personalized to achieve better clinical outcomes. To minimize the risk of hyperkalemia, clinicians should be aware of CKD as well as a decline in kidney function due to dehydration, particularly in elderly patients. Additional studies are warranted to develop a monitoring protocol for potassium levels and preemptive medicine utilizing pharmacometric models, considering drug–drug interactions using a model-based approach.

## Figures and Tables

**Table 1:** Clinical evidence for mineralocorticoid receptor antagonists.

Trial	Population	Comparator	Efficacy	Safety
Spironolactone				
RALES	HFrEF	Placebo	Mortality RR = 0.70, 95% CI: 0.60–0.82	Gynecomastia↑
TOPCAT	HFpEF	Placebo	Mortality HR = 0.91, 95% CI: 0.77–1.08	Hyperkalemia↑
BARACK-D	Older age + CKD	Standard care	Mortality HR = 1.09, 95% CI: 0.70–1.70	Hyperkalemia↑
Eplerenone				
EMPHASIS-HF	HFrEF	Placebo	Mortality HR = 0.76, 95% CI: 0.62–0.93	Hyperkalemia↑
EPHESUS	HFrEF	Placebo	Mortality HR = 0.85, 95% CI: 0.75–0.96	Hyperkalemia↑
Finerenone				
FIDELIO-DKD	T2DM + CKD	Placebo	CKD progression HR = 0.82, 95% CI: 0.73–0.93	Hyperkalemia↑
FIGARO-DKD	T2DM + CKD	Placebo	Cardiovascular events HR = 0.87, 95% CI: 0.76–0.98	Hyperkalemia↑
FINEARTS-HF	HFpEF	Placebo	Cardiovascular death HR = 0.84, 95% CI: 0.74–0.95	Hyperkalemia↑
Esaxerenone				
ESAX-HTN	HT	Eplerenone	Blood pressure↓	Hyperkalemia↔
EAGLE-DH	T2DM + HT	None	Blood pressure↓ and urinary protein↓	Hyperkalemia↔

Abbreviations: HFrEF, heart failure with reduced ejection fraction; HFpEF, heart failure with preserved ejection fraction; CKD, chronic kidney disease; T2DM, type 2 diabetes mellitus; HT, hypertension; RR, relative risk; HR, hazard ratio; 95% CI, 95% confidence interval. The up and down arrows indicate an increase and decrease in outcomes, respectively.

**Table 2:** Pharmacokinetics of mineralocorticoid receptor antagonists.

	F, %	Ae, %	f_b_, %	Vd, L	CL, L/h	Metabolism
Spironolactone	25	2.9	>89	96.8	18.1	non-CYP
Eplerenone	69	2.5	49.4	45.5 *	5.6 *	CYP3A4
Finerenone	43.5	0.825	91.7	52.6	22.3	CYP2C8 and CYP3A4
Esaxerenone	90.8	1.6	98.2 to 99.0	80	3.7	CYP3A4/5

Abbreviations: F, absolute bioavailability; Ae, urinary excretion ratio; f_b_, protein-binding ratio; Vd, volume distribution; CL, total clearance; CYP, cytochrome P450. *: Apparent values. The numerical parameters for spironolactone indicated the presence of canrenone (an active metabolite). References: spironolactone, [49,50,51,52]; eplerenone, [53,54]; finerenone, [55,56]; esaxerenone, [57,58].

**Table 3:** Mechanism of drug–drug interaction regarding mineralocorticoid receptor antagonists.

Mechanism	Precipitant Drug
Pharmacokinetics	
CYP3A4 inhibition *	CYP3A4 inhibitors (e.g., Ritonavir, Azole antifungals, Macrolides)
Pharmacodynamics	
Inhibition of renin release	NSAIDs, CNI, β blocker
RAAS inhibition	ACEi, ARB
ENaC inhibition	Trimethoprim

Abbreviations: CYP, cytochrome; NSAIDs, non-steroidal anti-inflammatory drugs; CNI, calcineurin inhibitor; RAAS, renin–angiotensin–aldosterone; ACEi, angiotensin-converting enzyme inhibitor; ARB, angiotensin-receptor blocker; ENaC, epithelial sodium channel. *: Eplerenone, finerenone, and esaxerenone.

## Data Availability

No new data were created or analyzed in this study.

## Abbreviations

The following abbreviations are used in this manuscript: CKD, chronic kidney disease; eGFR, estimated glomerular filtration rate; RAAS, renin–angiotensin–aldosterone; SGLT2i, sodium-glucose cotransporter 2 inhibitor; MRA, mineralocorticoid receptor antagonist; ACEi, angiotensin-converting enzyme inhibitor; LVEF, left ventricular ejection fraction; ARB, angiotensin-receptor blocker; IC_50_, half maximal inhibitory concentration; ENaC, epithelial sodium channel; Na-K ATPase, sodium-potassium ATPase; ROMK, renal outer medullary potassium channel; CYP, cytochrome; AUC, area under the plasma drug concentration-time curve; F, absolute bioavailability; CL_h_, hepatic clearance; f_u_, unbound fraction; CL_inth_, hepatic intrinsic clearance; AUC_f_, area under the unbound plasma drug concentration-time curve; NSAIDs, non-steroidal anti-inflammatory drugs.
